# The trophoblast surface becomes refractory to adhesion by congenitally transmitted *Toxoplasma gondii* and *Listeria monocytogenes* during cytotrophoblast to syncytiotrophoblast development

**DOI:** 10.1128/msphere.00748-23

**Published:** 2024-05-21

**Authors:** Rafaela J. da Silva, Leah F. Cabo, Jada L. George, Laty A. Cahoon, Liheng Yang, Carolyn B. Coyne, Jon P. Boyle

**Affiliations:** 1 Department of Biological Sciences, Dietrich School of Arts, University of Pittsburgh, Pittsburgh, Pennsylvania, USA; 2 Department of Integrative Immunobiology, Duke University School of Medicine, Durham, North Carolina, USA; University of Georgia, Athens, Georgia, USA

**Keywords:** human trophoblast stem cells, syncytiotrophoblast, cytotrophoblast, *Toxoplasma gondii*, transcriptome, *Listeria monocytogenes*

## Abstract

The placenta is a critical barrier against viral, bacterial, and eukaryotic pathogens. For most teratogenic pathogens, the precise molecular mechanisms of placental resistance are still being unraveled. Given the importance of understanding these mechanisms and challenges in replicating trophoblast-pathogen interactions using *in vitro* models, we tested an existing stem-cell-derived model of trophoblast development for its relevance to infection with *Toxoplasma gondii*. We grew human trophoblast stem cells (TS^CT^) under conditions leading to either syncytiotrophoblast (TS^SYN^) or cytotrophoblast (TS^CYT^) and infected them with *T. gondii*. We evaluated *T. gondii* proliferation and invasion, cell ultrastructure, as well as for transcriptome changes after infection. TS^SYNs^ cells showed similar ultrastructure compared to primary cells and villous explants when analyzed by transmission electron microscopy and scanning electron microscopy (SEM), a resistance to *T. gondii* adhesion could be visualized on the SEM level. Furthermore, TS^SYNs^ were highly refractory to parasite adhesion and replication, while TS^CYTs^ were not. RNA-seq data on mock-treated and infected cells identified differences between cell types as well as how they responded to *T. gondii* infection. We also evaluated if TS^SC^-derived SYNs and CYTs had distinct resistance profiles to another vertically transmitted facultative intracellular pathogen, *Listeria monocytogenes*. We demonstrate that TS^SYNs^ are highly resistant to *L. monocytogenes*, while TS^CYTs^ are not. Like *T. gondii*, TS^SYN^ resistance to *L. monocytogenes* was at the level of bacterial adhesion. Altogether, our data indicate that stem-cell-derived trophoblasts recapitulate resistance profiles of primary cells to *T. gondii* and highlight the critical importance of the placental surface in cell-autonomous resistance to teratogens.

**IMPORTANCE:**

Congenital toxoplasmosis can cause a devastating consequence to the fetus. To reach the fetus’s tissues, *Toxoplasma gondii* must cross the placenta barrier. However, how this parasite crosses the placenta and the precise molecular mechanisms of placental resistance to this parasite are still unknown. In this study, we aimed to characterize a new cellular model of human trophoblast stem cells to determine their resistance, susceptibility, and response to *T. gondii*. Syncytiotrophoblast derived from trophoblast stem cells recapitulate the resistance profile similarly to placenta cells. We also showed that these cells are highly resistant to *Listeria monocytogenes*, at the level of bacterial adhesion. Our results suggest that resisting pathogen adhesion/attachment may be a generalized mechanism of syncytiotrophoblast resistance, and trophoblast stem cells represent a promising model to investigate cell-intrinsic mechanisms of resistance to pathogen adhesion and replication.

## INTRODUCTION

Congenital infection occurs when a fetus contracts an infection from the mother during pregnancy. The impact on the developing fetus can vary depending upon factors such as the gestational age during the infection and the specific pathogen responsible, resulting in a wide array of outcomes including miscarriage, stillbirth, fetal malformation, and neonatal death. Pathogens such as *Toxoplasma gondii and Listeria monocytogenes* (*Lm*) are important among the major causes of congenital infections and are related to several adverse fetal and neonatal outcomes (*1, 2*).

*T. gondii* is an obligatory intracellular protozoan parasite responsible for the clinical illness toxoplasmosis and is particularly important as a causative agent of disease in immunocompromised and pregnant individuals (3, 4). In immunocompetent patients, toxoplasmosis is generally asymptomatic (5). However, congenital toxoplasmosis, whereby an immunocompetent mother transmits the parasite to their developing fetus, can be lethal (6, 7). Congenital toxoplasmosis is one of the most severe forms of the disease with primary infection during pregnancy resulting in miscarriage, stillbirth, premature birth, malformations, and neurological and/or ocular disorders in newborns (4, 8–10).

To reach the fetus and cause congenital toxoplasmosis, *T. gondii* must cross the barriers protecting the fetus, including the placenta (6, 7, 11). This organ is the primary site of nutrient, and gas exchange between mother and fetus and *T. gondii* is capable of broad dissemination within the host via the bloodstream, highlighting the importance of encounters between *T. gondii* and the placenta. The placenta also produces hormones and functions as an immunological and physical barrier to bloodborne pathogens (2, 12–14). *T. gondii* infection of the fetus is not the rule and occurs approximately in 40% of pregnant women who are infected for the first time during gestation (15). It is likely, but yet unproven, that the placenta protects the fetus from infection in at least some of these cases.

Structurally, the placenta is formed by villous trees that are either free-floating and bathed in maternal blood or anchored in the decidua. The inner layer of each villous tree is composed of cytotrophoblast (CYT). CYT are mononucleated cells that are responsible for (i) replenishing and growing the protective syncytiotrophoblast (SYN) layer via cell fusion and (ii) differentiating into extravillous trophoblast cells (EVT) (2, 16). The SYN layer is made up of a multinucleated cell that is bathed in maternal blood and present on the outermost surface of a floating villous (2, 17, 18). In contrast, EVTs are mononucleated, mesenchymal cells with an invasive profile that anchor the placenta in the decidua, where they then interface with maternal decidual and immune cells (19, 20). The SYN layer is a critical component of fetal defense and, in recent years, has been found to be naturally pathogen resistant, including to viral pathogens like Zika virus (21), bacterial pathogens like *L. monocytogenes* (22, 23), and parasites like *T. gondii* (18, 24). Our prior work with *T. gondii* using primary human trophoblasts (PHTs) (18) has shown that SYNs resist *T. gondii* infection by (i) being refractory to parasite adhesion and (ii) restricting parasite replication and/or being parasiticidal (18, 24, 25). In contrast, and like nearly all other cell types studied to date, CYTs and EVTs are both susceptible to *T. gondii* infection (24).

The intrinsic mechanisms involved in restricting pathogen growth and invasion by SYN and mechanisms related to the susceptibility of CYT cells to the parasite are poorly understood. For *T. gondii*, SYNs represent one of the only known cell types that resist *T. gondii* adhesion and restrict its replication without treatment with interferon-γ, as this parasite is capable of infecting and thriving within most nucleated cells. *In vitro* models that faithfully replicate CYT, SYN, and EVT biology are critical for understanding these processes on the molecular level. While lineages of immortalized trophoblast cells derived from choriocarcinomas are often used, including BeWo, JEG-3, and JAR, even when they are syncytialized, they do not reproduce the sensitivity of primary trophoblast cells or villous explants (18, 25). While primary trophoblast cells can differentiate spontaneously into SYN, they present challenges of their own in that they are difficult to manipulate genetically due to their short lifespan *in vitro* (26).

In order to explore different cellular models to study the placenta cells and pathogens interactions, we were interested in characterizing and utilizing the human trophoblast stem cells (TS^CT^), previously isolated and described by Okae and collaborators (27) as a model to elucidate the differential susceptibility to *Toxoplasma* infection. Here, we investigate the utility of TS cells to study the genetics of resistance and susceptibility between SYN and CYT to *T. gondii* and find that they faithfully recapitulate the resistance profile of primary cells to both *T. gondii* and *L. monocytogenes*. We also find that they have important limitations with respect to their constitutive production of cytokines like interferon-λ and their ability to respond to *T. gondii* infection by the production of CCL22 (18, 28).

## MATERIALS AND METHODS

### Culture of human TS^CT^

TS^CTs^ (clone 27), derived from first-trimester placental tissue, were kindly provided by Professor Okae from Tohoku University, Japan. Cells from this line were cultured as described previously (27). Briefly, 75 cm^2^ flasks were incubated in TS medium containing 2 µg/mL iMatrix-55 (AMSBIO, Abingdon, UK) for 10 min at 37°C in 5% CO_2_. TS medium consist of basal medium (DMEM/F12; Gibco, Waltham, MA, USA), 1% ITS-X100 (ThermoFisher Scientific, Waltham, MA, USA), 0.3% acid fatty free bovine serum albumin (BSA) (Sigma, St. Louis, MO, USA), 200 µM of ascorbic acid (Sigma), 0.5% penicillin-streptomycin (ThermoFisher Scientific), and 0.5% of Knockout Serum Replacement (KSR) (Gibco), supplemented with 25 ng/ epidermal growth factor (EGF) (ThermoFisher Scientific), 2 µM CHIR99021 (Stemolecule Reprocell USA, Inc., Beltsville, MD, USA), 5 µM A83-01 (Stemolecule Reprocell USA, Inc., Beltsville, MD, USA), 0.8 mM VPA (APExBIO, Houston, TX, USA), and 5 µM Y27632 (Stemolecule Reprocell USA, Inc., Beltsville, MD, USA). Later, cells were seeded in a ratio of 1:3 and incubated at 37°C and 5% CO_2_, until they reached 80% confluency. After that, cells were collected using TrypLE (Sigma) for 10 min at 37°C and passaged to a new pre-coated flask. To keep the cells, we used all the reagents listed above. However, when we performed the infection with *T. gondii*, we used TS medium lacking the A83-01 inhibitor.

### Differentiation of cytotrophoblast cells into syncytiotrophoblast cells and *T. gondii* infection

To induce syncytiotrophoblast (TS^SYN^) development from cytotrophoblast cells (TS^CYT^), we used both differentiation protocols as outlined in reference 27, with minor modifications. Briefly, for a mixed population of CYTs and SYNs, TS basal medium was supplemented with 5 µM Y27632 (Stemolecule Reprocell USA, Inc.), while for pure populations of TS^SYN^, we used ST (3D) medium (DMEM/F12 [Gibco], ITS-X100 [ThermoFisher Scientific], 0.3% fatty acid-free BSA [Sigma], 0.5% penicillin-streptomycin [ThermoFisher Scientific], 0.1 mM 2-mercaptoethanol [Fisher Scientific]), supplemented with 2.5 µM Y27632 (Stemolecule Reprocell USA), 2 µM Forskolin (Sigma), 4% EGF (ThermoFisher Scientific), and 50 ng/mL KSR (Gibco). In both cases, the medium was added in six-well plates for 10 min at 37°C, and cells were seeded in a ratio of 1.5 × 10^5^ in six-well plates. For mixed populations, the medium was replaced on day 3. For TS^SYN^ culture, each well was supplemented with ~2 mL of ST (3D) media on day 3, and the media was replaced on day 5 with ST (3D) media lacking forskolin for *T. gondii* infection.

### *T. gondii* culture

*T. gondii* strain RH (expressing YFP off of the GRA1 promoter) (18) was cultured in human foreskin fibroblast (HFF) in complete Dulbecco’s modified Eagle’s medium (cDMEM; ThermoFisher Scientific plus 100 U/mL penicillin/streptomycin, 100 µg/mL streptomycin, 2 mM L-glutamine, 10% fetal bovine serum(FBS), and 3.7 g NaH2CO3/L) and incubated in 5% CO_2_ and 37°C. For infection assays, infected monolayers were scraped and syringe-lysed to release tachyzoites, and then, parasites were pelleted at 800 × *g* for 10 min. For mock infections, the same parasite preparations were passed through a 0.22 µm filter (Millipore, Burlington, MA, USA), and the eluate was used to treat host cells at the same dilution as the parasite preparation.

### *L. monocytogenes* strains and culture

We used the following *L. monocytogenes* strains: strain 10403S served as the wild-type strain along with isogenic Δ*prsA2* (NF-L1651) (29) Δ*hly* (DP-L2161) (29) strains with deletions in the *prsA2* and *hly* genes, respectively. We also used *Lm*-GFP (DP-L4092) for imaging *L. monocytogenes* interactions with placental cells (30). For invasion assays, bacteria were grown overnight without agitation at 37°C in brain heart infusion broth (BHI; Oxoid) until an OD_600_ of 0.7–0.9 was reached. Bacteria were washed twice and suspended in a cell culture medium without serum and antibiotics for infection experiments.

### General *T. gondii* infection and antibody staining protocol

Trophoblast stem cells grown under different conditions described above were seeded in a ratio of 1.5 × 10^5^ in a 6-well plate, infected, or mock-infected with *T. gondii* RH:YFP. TS^CYT^ was induced to form TS^SYN^ for 4–5 days and then infected with *T. gondii* at different multiplicities of infection(MOIs) depending on the experiment. Cells were collected and evaluated for 24 and 48 h post-infection. For most of 24 h infections, TS^SYNs^ were infected on day 5 of differentiation at an MOI of 5 parasites based on the number of cells plated on the first day of the experiment. In both cases, the TS^CYT^ were infected 1 day after plating, and the mixed population of trophoblasts was infected on day 3 of differentiation. Cell passages were staggered so that the same parasite preparation could be used to infect TS^SYN^ and TS^CYT^ simultaneously.

To evaluate the difference in growth of *T. gondii* over time in TS^SYNs^ and TS^CYTs^, cells were infected on day 4 of differentiation of TS^SYNs^ with MOI of 1.5 parasites. Cells were collected after 24 h and 48 h post-infection and processed for immunofluorescence. The vacuole size (μm) of the parasite was quantified in each cell area using ImageJ software, and the data were expressed as the average of each vacuole size/cell area in each field of view at each time point. In TS^SYNs^ samples, the vacuole size was only measured in SDC1-positive cells. Twelve images were taken across three different coverslips using epifluorescence microscopy and analyzed using ImageJ software.

To evaluate on which day of differentiation TS^SYN^ becomes resistant to *T. gondii* infection, TS^SYN^ cells were plated in six-well plated in duplicate and infected with the parasite every day from days 1 to 6. The parasites were allowed to grow for 24 h for each time point, and then, the cells were fixed and processed for immunofluorescence. Due to a mixed population of cells during the differentiation process, in different time points, the data were quantified as a total vacuole size of *T. gondii*/total of cell area in each field of view using ImageJ. The total parasite area was measured in 10 fields of view.

For immunofluorescence assay, cells were fixed using 4% paraformaldehyde (PFA; ThermoFisher Scientific) for 12 min, rinsed with phosphate buffered saline (PBS) (ThermoFisher Scientific), and permeabilized with 0.2% Triton X-100 in PBS for 10 min. Cells were then incubated with the TS^SYN^ marker anti-syndecan-1 (SDC-1; 1:500; ab128936, Abcam, Cambridge, UK) and TS^CYT^ marker, anti-integrin alpha-6 (ITGA6; 1:1,000; MA5-16884, ThermoFisher Scientific) for 1 h at room temperature. Alexa Fluor 594 and 647-conjugated (A-21209 and A-32733, Life Technologies Alexa Fluor H+L, Carlsbad, CA, USA) were used as secondary antibodies for 45 min. All images were taken using the Olympus IX83 epifluorescence microscope and X-Cite 120 LED Boost camera, at 20× or 40× objective lens.

### *L. monocytogenes* infection and gentamicin survival assay

TS^SYNs^ and TS^CYTs^ were cultured in six-well plates and *L. monocytogenes* wildtype, and Δ*prsA2* or Δ*hly* was used to infect cells in triplicate at 8 × 10^6^ bacteria per well. As a control, a mock infection was performed using 0.2-μm-filtered *L. monocytogenes* culture supernatant. Inoculated cells were incubated for 1 h at 37°C with 5% CO_2_. Cells were then washed with fresh cell culture media and incubated for 7 h with gentamicin (5 µg/mL). Two washes were performed with 1× PBS, and cells were lysed by incubation with 0.25% Triton X-100 for 5 min at room temperature (RT) followed by plating of serial dilutions of the cell lysates on BHI agar plates. Plates were incubated at 37°C for 36 h after which bacteria were enumerated and colony-forming units were calculated. TS^SYN^ and TS^CYT^ cells were also collected for immunofluorescence assay as described above and for TEM. For immunofluorescence, all photos were taken using the Nikon A1R-HD TIRF confocal microscope and Photometrics PRIME BS1 camera.

### qPCR

To quantify the number of parasites in TS^SYN^ and TS^CYT^, cells were infected with *T. gondii* at an MOI of 5 parasites for 24 h. After that, genomic DNA was extracted using the GeneJet Genomic DNA Purification kit following the manufacturer’s instructions (TermoFisher Scientific). qPCR was used to quantify the total number of genomes using primers targeting the *T. gondii* GRA1 gene and primers targeting human β-ACTIN as a control gene. All reactions were performed in triplicate using a QuantStudio 3 Real-Time PCR System (ThermoFisher). The DNA was mixed with SYBR Green buffer (BioRad, Hercules, CA, USA) and 1 µL (5 µM) of both forward and reverse primers and ddH_2_O. Genes were amplified using a standard protocol (95°C for 10 min and 40 cycles of 95°C for 15 s and 60°C for 1 min), and data were analyzed with QuantStudio Design & Analysis Software. To determine the total number of parasite genomes, a standard curve of known parasite numbers ranging from 1 × 10^7^ to 1 × 10^1^ was also performed using *T. gondii* GRA1 primers: *T. gondii* GRA1 forward, TTAACGTGGAGGAGGTGATTG; GRA1 reverse, TCCTCTACTGTTTCGCCTTTG, human β-ACTIN forward, GCGAGAAGATGACCCAGATC; human β-ACTIN reverse, CCAGTGGTACGGCCAGAGG. Two experiments in triplicate were performed.

To quantify the expression level of IL-1β in cells infected and mock-infected with *L. monocytogenes*, the RNA was extracted using the Qiagen RNA extraction kit following the manufacturer’s instructions (Qiagen, Hilden, Germany). RNA was analyzed by gel electrophoresis and quantified by Nanodrop. cDNA was generated from 0.5 µg of RNA using the SuperScript IV First-Strand synthesis system (ThermoFisher Scientific). IL-1β forward: CTCTCACCTCTCCTACTCACTT; IL-1β reverse: TCAGAATGTGGGAGCGAATG. β-ACTIN was used as a reference gene. DeltaCt values (query gene Ct – control gene Ct) were used for statistical comparisons and then converted to fold-difference using the 2^-ΔΔCt^ method. One experiment in triplicate was performed.

### Transcriptional analysis of TS^CYT^ and TS^SYN^ using RNA-seq

TS^SYN^ and TS^CYT^ cells were seeded and infected in the same conditions as described above for qPCR, and RNA was extracted to perform RNA-seq. Strand-specific, oligo-dT-generated sequencing libraries were prepared at Core Facility at the University of Pittsburgh, and 2 × 66 bp reads were sequenced on a NextSeq 2000 (Illumina). Read libraries were mapped to the human (hg38) transcriptomes using CLC Genomics Workbench v.23.0. Raw read counts were analyzed using the DESeq2 package implemented in R (31) using default settings to identify transcripts of significantly different abundance. These data were also used to calculate relative log2-fold change values across cell type and infection parameter, and these data were fed into pre-ranked Gene Set Enrichment Analysis (GSEA) to identify host gene sets that were negatively or positively enriched. One experiment with three replicates was performed.

### Luminex assay

To evaluate the cytokine and chemokine profile secreted by TS^CYT^ and TS^SYN^ infected or mock-infected with *T. gondii*, supernatants were collected from those cells Luminex assay was performed using the following kits according to the manufacturer’s instructions: Bio-Plex Pro Human Inflammation Panel 1, 37-Plex kit (171AL001M; Bio-Rad) Bio-Plex Human Chemokine Panel, 40-plex (171AK99MR2; Bio-Rad).

In order to verify the CCL22 production in response to the infection with *T. gondii* in a different system to compare to our data with TS cells, we performed an experiment using trophoblast organoids (line TO74) (32). TOs were cultured as described previously (32), and for infection, they were removed from the Matrigel “dome” and maintained in suspension. Then, TOs were infected with 1 × 10^6^ *T. gondii* tachyzoites (RH-YFP) for 24 h. As a control, TOs were mock-infected with the parasite. The supernatants from infected and control conditions were collected, and the levels of C-C motif chemokine 22 (CCL22), macrophage migration inhibitory factor (MIF), and osteopontin (OPN) were measured by Luminex assay.

### Transmission and scanning microscopy

For transmission electron microscopy (TEM), TS^CYTs^ and TS^SYNs^ were uninfected or infected with *L. monocytogenes* wildtype for 8 h. TS^CYTs^ were fixed with 2.5% glutaraldehyde in PBS for 1 h at room temperature and washed once with PBS. Meanwhile, TS^SYNs^ were pelleted, and the samples were kept in 2.5% glutaraldehyde. They were processed by the Center of Biological Imagining-CBI at the University of Pittsburgh. Briefly, the samples were incubated for 1 h at 4°C in 1% OsO_4_ with 1% potassium ferricyanide and washed three times with 1× PBS. Then, they were dehydrated in a graded series of alcohol for 10 min with three changes in 100% ethanol for 15 min and changed three times in epon for 1 h each. Following the removal of epon, samples were covered with resin and polymerized at 37°C overnight and then 48 h at 60°C. After the samples were cross sectioned, they were imaged using the JEOL 1400-Plus microscope.

For scanning electron microscopy (SEM), TS^CYTs^ and TS^SYNs^ were infected with *T. gondii* for 2 h, washed twice with PBS, and the samples were fixed as described above and processed by the CBI facility. Images were taken using the JSM-6335F microscope.

### Statistical analysis

Statistical analyses (besides those on RNA-seq data which were described above) were performed using GraphPad Prism 9.0 (La Jolla, CA, USA). Differences between conditions were assessed by one-way analysis of variance (one-way ANOVA) followed by the Bonferroni multiple comparison *post-hoc* test for parametric samples, or Kruskal Wallis followed by Dunn’s multiple comparisons for a non-parametric, by two-way ANOVA, or by *t* test for parametric samples or Mann-Whitney for non-parametric, if comparing only two groups.

## RESULTS

### Syncytiotrophoblasts are more resistant to *T. gondii* infection than cytotrophoblasts

**Fig 1:**
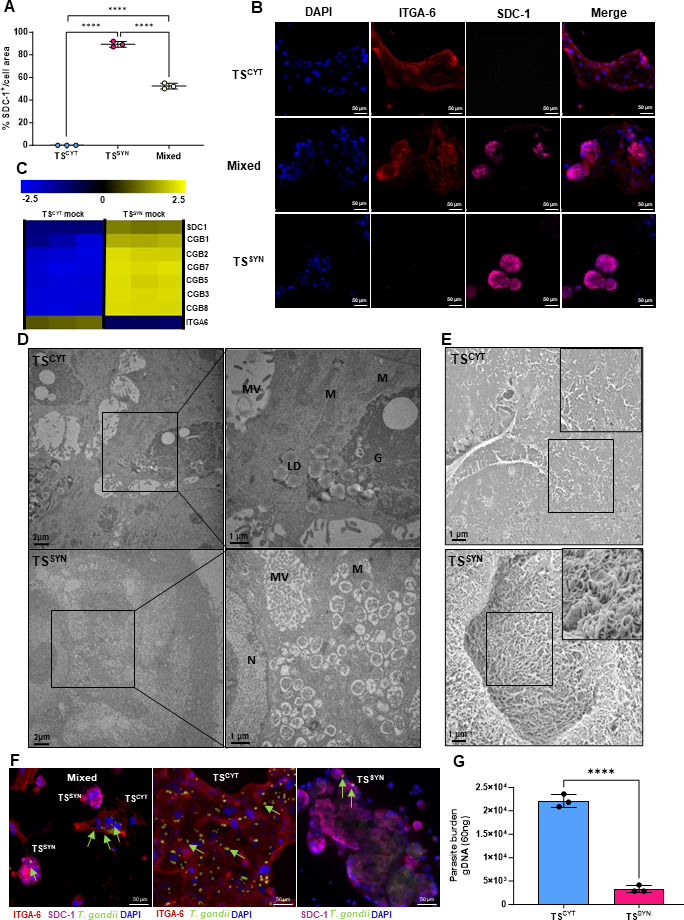
TS cells differentiated into syncytiotrophoblast-like cells have innate resistance to *T. gondii*. TS^CYT^, TS^SYN^, and mixed populations of TS^SYNs^ and TS^CYTs^ were seeded in a ratio of 1.5 × 10^5^ in a 6-well plate in triplicate. Cells were infected or not with *T. gondii* with MOI of 5 for 24 h prior to immunofluorescence, qPCR, or RNA-seq analysis. Immunofluorescence was performed to stain cells with TS^SYN^ marker (SDC-1) (pink), TS^CYT^ marker (ITGA-6; red), DAPI (4′,6-diamidino-2-phenylindole) (blue), and *T. gondii* (green). (**A**) Quantification of SDC-1 in TS^CYT^, TS^SYN^, and mixed populations of TS^SYNs^ and TS^CYTs^ was calculated by the percentage (%) of fluorescence expression of SDC-1 normalized by total cell area. Differences between cells were analyzed by one-way ANOVA with Bonferroni multiple comparison *post-hoc* test. ^****^P < 0.0001. (**B**) Representative images showing immunofluorescence microscopy of TS^CYT^, mixed populations of TS^SYN^ and TS^CYT^, and 3D TS^SYN^ (which exclusively form SYNs). Images such as these were used to generate the data in panel A. (**C**) Heat map showing transcript abundance in TS^CYT^ and TS^SYN^ for ITGA-6, CGB1, 2, 7, 3, 5, 8, and SDC-1 in mock-treated and infected cells (*Padj* 0.01; log fold change ≥ 2 or ≤ −2 for all genes shown). These data provide transcriptional evidence for the establishment of TS^CYT^ and TS^SYN^ cultures in our laboratory, consistent with prior work (27). (**D**) TEM photos showing the ultrastructure TS^CYTs^, highlighting the presence of lipid droplets (LD), glycogen granules (**G**), microvilli (MV), and mitochondria (**M**), and in TS^SYNs^, we observed the presence of nuclei (**N**), mitochondria (MV), and a dense microvilli (MV). (**E**) SEM showing the difference in the density of microvilli in TS^CYTs^ and TS^SYNs^. (**F**) Immunofluorescence images demonstrating differences in *T. gondii* (RH:YFP; green) infection and proliferation between TS^CYT^ and TS^SYN^ in mixed population, TS^CYT^ culture, and TS^SYN^ 3D condition. Green arrows indicate parasites that are either inside or associated with the outside of host cells. (**E**) Quantifying *T. gondii* burden using qPCR for the GRA1 transcript utilizing the standard curve. Differences between TS^CYT^ and TS^SYN^ were analyzed by *t* test, ^****^P = 0.001. Photos were taken using 20× magnification. Scale bar: 50 µm.

TS^CYT^ cells were cultured and differentiated into TS^SYN^ cells as described previously (27), and we quantified the success of differentiation using the TS^SYN^ marker SDC-1 (27) and the TS^CYT^ marker ITGA-6. We found that cells in “CYT” conditions were negative for SDC-1, as expected (Fig. 1A and B), while ~90% of those cells cultured in 3D SYN conditions expressed SDC-1 and, therefore, were likely TS^SYNs^ (Fig. 1A and B). When the TS medium was supplemented only with Y27632, this resulted in a mixed population of ~45% TS^CYT^- and 55% TS^SYN^-like cells (Fig. 1A and B). These data indicated that we were able to recapitulate the differentiation procedure described previously (27).

To further validate the differentiation process in our laboratory, we used RNA-seq and first focused on transcript levels for SYN markers (CGB1, CGB2, CGB7, CGB5, CGB3, CGB8, and SDC-1) and the CYT marker ITGA-6. TS^SYN^ cells in 3D medium had significantly higher transcript abundance for SDC-1 and members of the *CGB* gene family and significantly lower levels of ITGA-6 (Fig. 1C), while TS^CYT^ cells had significantly higher levels of ITGA-6 transcript and significantly lower levels of SDC-1 and *CGB* family member transcripts (Fig. 1C). These data suggest that we were able to generate TS^SYN^ cells and indicated that 3D medium was the most efficient protocol to generate TS^SYN^ cells as previously described (27).

We also performed TEM and SEM to evaluate differences in ultrastructure between TS^CYTs^ and TS^SYNs^. Transmission electron microscopy revealed that TS^CYTs^ have large amounts of glycogen granules and lipid droplets scattered in the cytoplasm, while this is not seen in TS^SYNs^ (Fig. 1D). We also could observe the differences in mitochondria morphologically, similar to what is described by reference 33. TS^CYTs^ have larger mitochondria with a lamellar crista (Fig. S1A and B), while TS^SYNs^ have smaller mitochondria containing vesicular cristae (Fig. S1C and D). Scanning electron microscopy shows that the surface of TS^SYN^ is densely covered by microvilli, while microvilli, in TS^CYTs^, were much sparse (Fig. 1E). These findings indicate that TS^CYTs^ and TS^SYNs^ generated in our hands bear all of the expected characteristics.

Given the known resistance of SYNs to *T. gondii* infection compared to CYTs in both PHT cells and placental explants (18, 24), we used trophoblast stem cells to generate TS^SYNs^ and compare their sensitivity to *T. gondii* infection with TS^CYTs^. We generated pure TS^CYT^, TS^SYN^ (3D), and cultures of mixed TS^CYTs^ and TS^SYNs^ and infected them with RH:YFP parasites with an MOI of 5 for 24 h. Qualitatively, images of infected TS^CYTs^ and TS^SYNs^ show dramatic differences in *T. gondii* numbers that have infected each cell type, with TS^CYT^ cells being much more susceptible to parasite infection compared to TS^SYN^ (Fig. 1F). Using qPCR for the *T. gondii* gene *GRA1* as a proxy for parasite number, based on a standard curve, we found that the parasite burden was higher in TS^CYT^ cells compared to TS^SYN^ (*P* = 0.0001; Fig. 1G), further supporting the reduced number of *T. gondii* associated with TS^SYN^ compared to TS^CYT^. These data suggest that TS^SYNs^ are similarly resistant to *T. gondii* as those generated during the culture of placental explants and primary human trophoblast cells (18, 24).

### TS^SYNs^ become resistant on day 4 of differentiation

**Fig 2:**
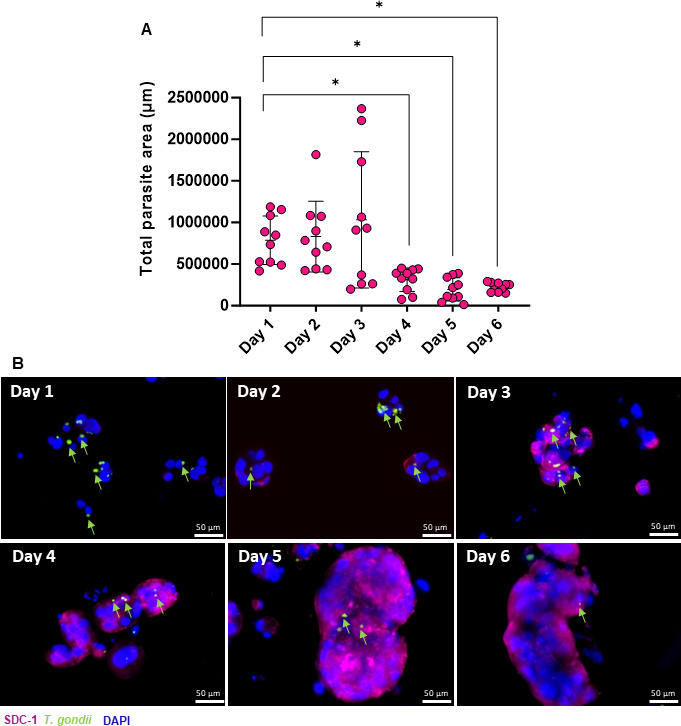
TS^SYNs^ become resistant to *T. gondii* on day 4 of differentiation. TS^SYNS^ differentiated in a ratio of 1.5 × 10^5^ in a 6-well plate in duplicate. Cells were infected with *T. gondii* in each time point on days 1 to 6 with MOI of 5, based on the number of cells plated on the first day of differentiation, and collected after 24 h post-infection, fixed with 4% PFA and then visualized using immunofluorescence. (**A**) The total of parasite area was measured in each field of view by ImageJ software. The data were collected in 10 fields of view for each time point. Differences between the days of differentiation and the total of parasite vacuole size were analyzed by one-way ANOVA with Bonferroni multiple comparison *post-hoc* test. (**B**) Immunofluorescence images illustrate the parasite growth in different days of differentiation from days 1 to 6. SDC-1^+^ (TS^SYN^) in pink, *T. gondii* in green, and DAPI in blue. Green arrows indicate parasites inside the host cells. Photos were taken using 20× magnification. Scale bar: 50 µm.

To generate a pure population of TS^SYNs^, we induce the differentiation for 6 days using the ST 3D medium. Given that, we want to evaluate on which day of differentiation the cells would become resistant to *T. gondii* infection. Cells were plated in duplicate and infected from day 1, the same day they were plated, and on the following days until day 6. Cells were infected with MOI of 5 in all conditions, based on the number of cells plated on the first day of differentiation. Cells were collected after 24 h of infection, and the total of vacuoles size was calculated per total cell area in a field of view. Our data show that from days 1 to 3 of differentiation, the cells are still susceptible to infection, and the parasite is able to proliferate intracellularly. However, from day 4, the cells become resistant compared to day 1 (*P* = 0.03; Fig. 2A). There is no significant difference between days 4, 5, and 6. Representative images show the progression of the differentiation with the SDC-1 staining and the formation of the multinucleated cells over time. We also can see the large number of *T. gondii* vacuoles in the cells on day 1 of differentiation when compared to days 4, 5, and 6 (Fig. 2B).

### *T. gondii* growth is restricted in TS^SYN^

**Fig 3:**
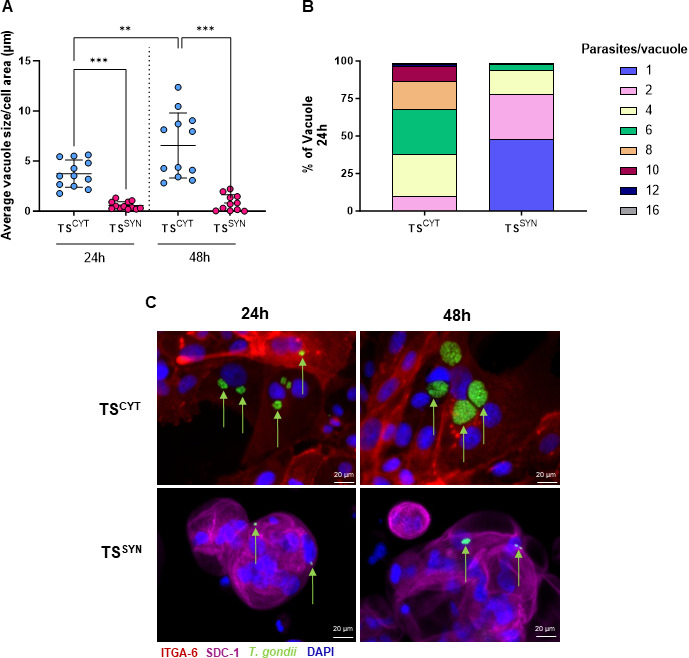
*T. gondii* growth is restricted in TS^SYN^ compared to TS^CYT^. TS^CYT^ and TS^SYN^ cells cultured from1.5 × 10^5^ cells in a 6-well plate and infected on days 3 and 5 post-plating, respectively, at an MOI of 1.5 for 24 h and 48 h. Cells were fixed with 4% PFA and then visualized using immunofluorescence. (**A**) *T. gondii* burden in TS^CYT^ and TS^SYN^ was measured based on the average vacuole size found in each cell and normalized by each host cell area at each time point. Twelve images were taken across three different coverslips using epifluorescence microscopy and analyzed using ImageJ software. (**B**) The rate of intracellular growth was also evaluated by counting the number of parasites per vacuole 24 h post-infection. A total of 80 vacuoles were counted over three different coverslips. (**C**) Immunofluorescence images illustrate the parasite growth in the distinct cell cultures at 24 and 48 h post-infection. ITGA-6^+^ cells (TS^CYT^) are shown in red, SDC-1^+^ (TS^SYN^) in pink, *T. gondii* in green, and DAPI in blue. Green arrows indicate parasites that are either inside or associated with the outside of host cells. Even after 48 h, there were very few parasites associated with the TS^SYNs^. Differences between TS^CYT^ and TS^SYN^ at different time points were analyzed by one-way ANOVA with a Bonferroni multiple comparison *post-hoc* test. ^*^P < 0.0001 when comparing TS^SYN^ and TS^CYT^ at each time point and when comparing across time points in TS^CYT^. Two experiments were performed with three replicates. Photos were taken using 40× magnification. Scale bar: 20 µm.

A limitation of PHT cells is their short (2–4 days) cultivation time *in vitro*, making long-term quantification of *T. gondii* growth very challenging. Given the longer survival times of TS^SYNs^ derived from trophoblast stem cells, we used this model to assess parasite growth in TS^SYNs^ and TS^CYTs^ over a 48-h period. To do this, we infected TS^CYT^ and TS^SYN^ with *T. gondii* at an MOI of 1.5 and quantified parasite abundance using immunofluorescence at 24 and 48 h post-infection. As expected, *T. gondii* numbers significantly increased at each time point in TS^CYT^ cells (*P* < 0.0021; Fig. 3A and C) and had significantly higher numbers of parasites compared to TS^SYNs^ at each time point (Fig. 3A and C). In contrast, parasite numbers did not significantly change over the course of the experiment in TS^SYNs^, suggesting that, similar to PHT cells (18), TS^SYNs^ restrict *T. gondii* replication (Fig. 3A and C). In addition to measuring the vacuole size, we also addressed the replication rate of the parasite by counting the number of parasites in 80 vacuoles across three different coverslips. Our data show again that the replication rate to *T. gondii* in TS^CYTs^ is much higher if compared with parasites that grow in TS^SYNs^ (Fig. 3B), while 30% or 27.5% of the vacuole have six or four, respectively, in TS^CYTs^, during the same time point, 47.5% of the vacuoles in TS^SYN^ have only one parasite. These data prove one more time the efficiency of these cells in controlling the *T. gondii* replication.

### TS^SYNs^ recapitulate the SYN-specific resistance of primary cells to *T. gondii* invasion

**Fig 4:**
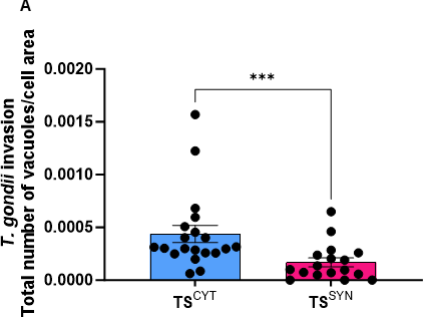
TS^SYN^ are less susceptible to *T. gondii* invasion compared to TS^CYT^. TS^CYT^ and TS^SYN^ were cultured on glass slides in 24-well plates and infected on days 3 and 5 post-culture, respectively, with an MOI of 5 for 24 h. Cells were fixed with 4% PFA, and the immunofluorescence was performed. (**A**) Quantification of *T. gondii* invasion in TS^CYT^ and TS^SYN^ was calculated by the total number of vacuoles with greater than one parasite per total cell area per field of view (differences between TS^CYT^ and TS^SYN^ were calculated by Mann-Whitney, ^***^P = 0.0004). Two independent experiments with three replicates were performed.

**Fig 5:**
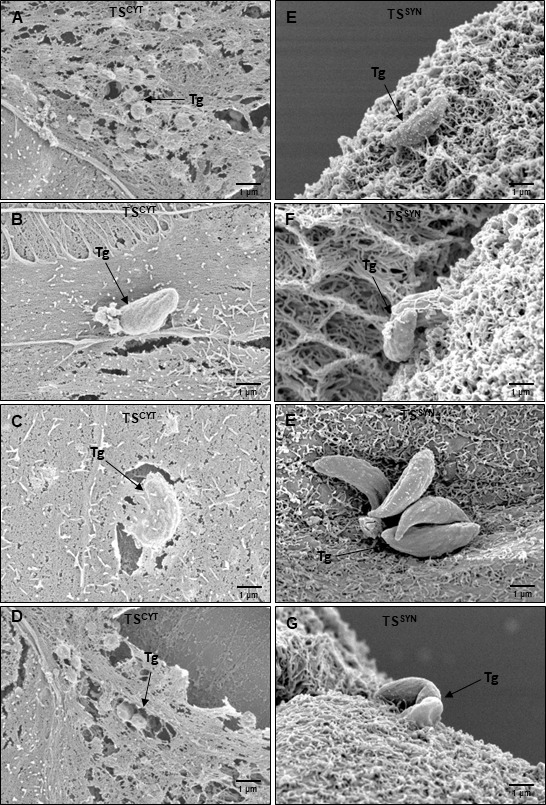
SEM in TS^CYTs^ and TS^SYN^ infected with *T. gondii*. TS^CYT^ and TS^SYN^ were infected for 2 h, and samples were collected for SEM. (**A–D**) qualitative images showing the parasite invasion process in TS^CYTs^, where we can visualize a large number of parasites under the membrane, suggesting a successful invasion process. (**E–G**) Qualitative images show parasites associated with TS^SYNs^. *T. gondii* = Tg. Black arrows indicate the parasite. Magnification: 10,000×, scale bar: 1 µm.

Since our prior work also established that SYNs from primary human trophoblasts were poorly invaded by *T. gondii*, we counted the total number of vacuoles present in each cell per field of view after 24 h post infection as a proxy of invasion rate in TS^CYTs^ and TS^SYNs^. A significantly higher number of parasite vacuoles were found in TS^CYT^ cells compared to TS^SYN^ cells per field of view (*P* = 0.0004; Fig. 4A), indicating that *T. gondii* invaded more TS^CYTs^ compared to TS^SYNs^. This phenotype recapitulates what was observed previously in PHT cells (18). Specifically, fewer parasites invaded TS^SYNs^ compared to TS^CYTs^. We also performed SEM in both cells after infection for 2 h in order to verify the interaction between *T. gondii* and the host cell membrane. Our qualitative data clearly show that lots of parasites invaded TS^CYTs^ (Fig. 5A through D), and in TS^SYNs^, *T. gondii* seems to be adhered to the membrane but rarely invades the cells in the same time frame as compared to TS^CYTs^ (Fig. 5E through G).

### TS^CYTs^ and TS^SYNs^ exhibit low production of immunoregulatory factors compared to PHTs

**Fig 6:**
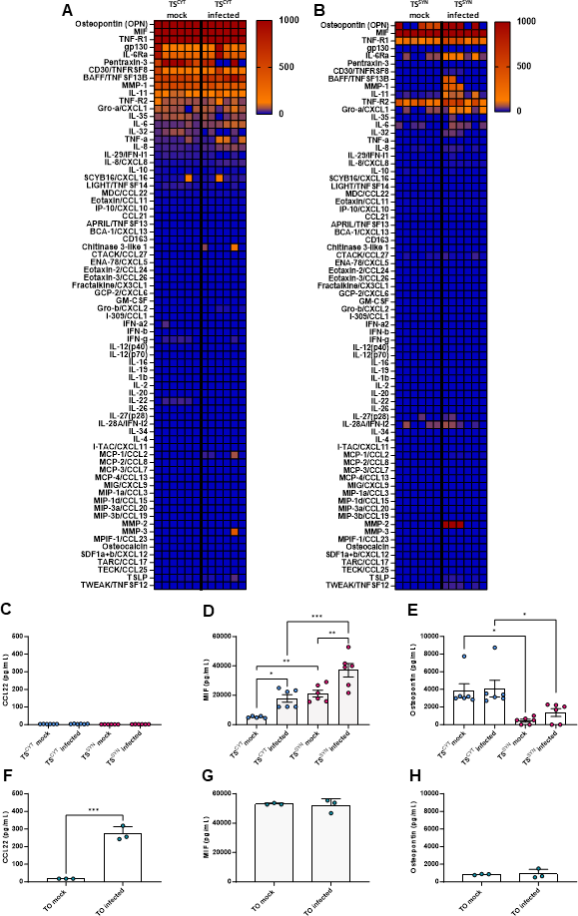
Cytokine quantification in supernatants from TS^SYN^ and TS^CYT^ and TOs mock-infected or infected with *T. gondii.* The supernatants of TS^CYT^, TS^SYN^, and TOs of mock or infected with *T. gondii* were collected after 24 h post-infection, and the Luminex assay was performed to visualize the immunomodulatory profile in both cells and the induction of CCL22 of TOs. The heatmap graphs show the difference in secretome levels of different cytokines, chemokines, and immune factors in (**A**) TS^CYT^ mock or infected and (**B**) TS^SYN^ mock or infected with *T. gondii*. Differences among the secretion of CCL22 (**C**), MIF (**D**), and OPN (**E**) in both cell types mock and infected are represented. We also highlight the differences in secretion levels of CCL22 (**F**), MIF (**G**), and OPN (**H**) in TO74. The data were expressed in pg/mL. Differences between TS^CYT^ and TS^SYN^ and between TO74 mock and infected at the cytokine level were analyzed by one-way ANOVA with a Bonferroni multiple comparison *post-hoc* test.

In addition to differences in susceptibility to *T. gondii* compared to many other cell types, PHTs, human placental villous explants and trophoblast organoids produce different cytokines, chemokines, and growth factors that are important for pregnancy maintenance and fetus defense (2, 32). Previous work has shown that CCL22 is produced by PHTs and villous explants in response to *T. gondii* infection while other cell types such as HFFs do not (18). We quantified 77 chemokines and cytokines using multianalyte Luminex-based profiling in supernatants of mock-infected and infected cells to evaluate the immunomodulatory secretome of TS^CYTs^ and TS^SYNs^. Even though TS cells recapitulate the resistance profile to *T. gondii* infection, the cells do not recapitulate the immune response to infection observed in villous explants and PHT cells. In our data, we defined reliable secretion factors measured above 100 pg/mL as a baseline. We found that both TS^CYTs^ and TS^SYNs^ did not secrete CCL22 when infected with *T. gondii* (Fig. 6A through C). In contrast, when we infected TOs, we detected a clear induction of CCL22 secretion compared to mock-infected organoids (Fig. 6F). Our data also show that TS^CYTs^ in both conditions secrete cytokines such as MIF, IL-11, and gp130, and immulogical factors including TNFRSF8, pentraxin-3, and MMP-1, TNF-R1, and OPN (Fig. 6A). On the other hand, TS^SYNs^ only secrete detectable amounts of two soluble factors (OPN and TNF-R2; Fig. 6B) and one cytokine (MIF; Fig. 6B and D). Interestingly, we observed that the soluble factor OPN presents significant low levels of secretion in TS^SYNS^ in both conditions mock and infected cells when compared with TS^CYTs^ mock and infected (Fig. 6A, B, and E). However, in TO74, this model produces lower levels of OPN when compared to TS cells, and the infection does not alter the production of this soluble factor (Fig. 6H). MIF is highly produced by TS^CYTs^ and TS^SYNs^ in both conditions (Fig. 6A, B, and F), but the infection with *T. gondii* induces significantly higher levels of this cytokine compared to the mock conditions, and infected TS^SYNs^ also release more MIF when compared to TS^CYTs^ (Fig. 6A, B, and D). On the other hand, TO74 releases high levels of MIF, but there is no significant difference between the mock and infected conditions (Fig. 6G).

Overall, our data show that trophoblast stem cells do not release many of the immunomodulatory compounds that are secreted by PHTs, highlighting that these cells do not recapitulate the similar immunological profile of PHTs, placenta explants, and organoids. This model, then, allows us to separate immune effector and signaling molecule production from innate cellular resistance that develops during the transition from CYT to SYN.

### *T. gondii-*infected TS^SYN^ and TS^CYT^ have distinct transcriptomes

**Fig 7:**
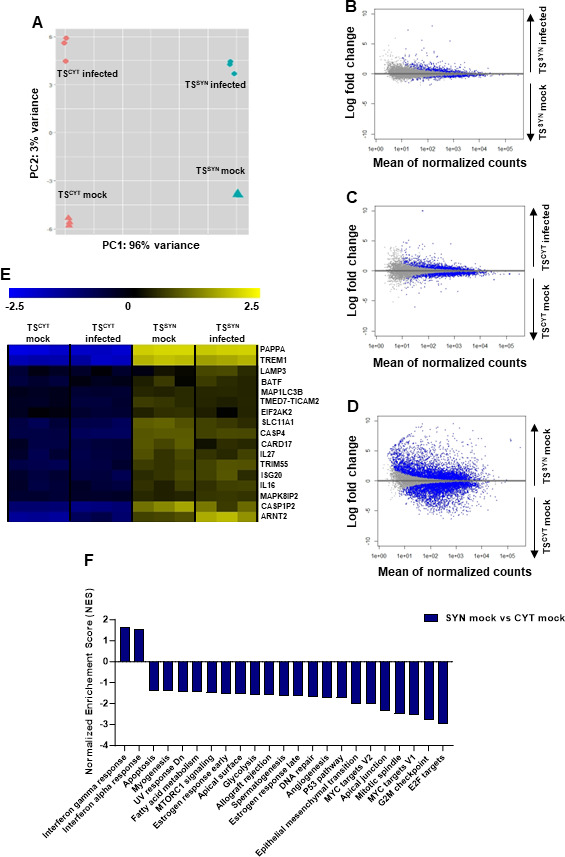
TS^SYN^ and TS^CYT^ infected cells reveal distinct gene expression profiles. Cells were cultured in 6-well plates and mock-treated or infected with *T. gondii* on days 3 and 6 post plating, respectively, with an MOI of 5 for 24 h. Cells were collected and processed for RNA-sequencing. (**A**) Principal components PC1 and PC2 of TS^CYT^ infected or mock-infected and TS^SYN^ infected or mock-infected differentiated samples along the cell type (PC1) or infection status (PC2) axes. (**B–D**) MA-plots of transcript abundance in TS^CYT^ and TS^SYN^ mock treated or infected with *T. gondii*. Blue dots represent genes of significantly different abundance based on the statistical comparison being performed: (**B**) TS^SYN^ infected vs TS^SYN^ mock; (**C**) TS^CYT^ infected vs TS^CYT^ mock; (**D**) TS^SYN^ mock vs TS^CYT^ mock. These plots illustrate that the most dramatic differences between the samples are driven by the cell type (TS^CYT^ or TS^SYN^). (**E**) Heat map showing immunity-related transcripts that were either constitutively different between cell types independent of infection status or altered in abundance by infection (*Padj <* 0.05; log fold change ≥ 1 or ≤ −1). We used normalized enrichment scores (NES) generated using pre-ranked GSEA from rlog-normalized data to evaluate the differences in enriched pathways between TS^SYN^ and TS^CYT^. (**F**) GSEA plot shows different gene set pathways related to metabolism in TS^SYN^ mock vs TS^CYT^ mock. For the graph, only significantly enriched pathways are shown (false discovery rate [FDR] *q*-value < 0.05). One experiment with three replicates was performed.

Another disadvantage of the PHT cell model is that the cultures are a mixed population of CYTs, SYNs, and also contain contaminating fibroblasts (18, 21, 34). The TS model described here provided a unique opportunity to observe the transcriptional response to infection in pure populations of cells that bear biological similarity to naturally occurring CYTs and SYNs (18, 27). After infecting TS^CYT^ and TS^SYN^ for 24 h with *T. gondii* RH:YFP, we performed strand-specific RNA-seq to compare the transcriptional responses of each of these cell types. We first used principal component analysis to broadly examine sample-by-sample differences in the presence and absence of *T. gondii*. Two major principal components were identified. PC1 encompassed 96% of the total variance with cell type (TS^SYN^ or TS^CYT^) varying primarily along this axis (Fig. 7A). The bulk of the remaining variance (3% out of 4%) was accounted for by infection state (mock or infected; Fig. 7A). These data confirm that TS^CYTs^ and TS^SYNs^ are transcriptionally distinct, as expected from prior transcriptional studies on these cell types (27). One surprise in these data was that the impact of infection on the TS^CYT^ and TS^SYN^ transcriptomes was similar between these cell types despite the dramatic difference in parasite infectivity (Fig. 1F, G and 3A, B). While we explore this further below by examining the specific sets of genes with altered transcript levels under each condition, this was a surprising result given that many of the well-known alterations in the host transcriptional profile require parasite invasion and/or parasite attachment to the host cell along with the secretion of host modulatory effectors (35, 36).

All transcripts and those of significantly different abundance based on DESeq2 analysis (*Padj* < 0.05; |log_2_fold difference| > 2) are shown in the MA-plots (log fold-difference vs average abundance for each transcript; Fig. 7B through D). As shown in Fig. 7B and C, MA plots comparing infection in both TS^SYN^ and TS^CYT^ have similar shapes and profiles, and but more significantly different transcript abundances are seen in TS^CYTs^ after infection, compared to TS^SYNs^. A unique feature of the SYN is its ability to resist *T. gondii* infection (18, 24) without prior exposure to activating cytokines like interferon γ. Given that TS^SYNs^ recapitulate this phenotype, we aimed to identify putative host resistance genes that might be either constitutively expressed in TS^SYNs^ compared to TS^CYTs^ and other susceptible cell types or induced by *T. gondii* infection uniquely in TS^SYNs^. Our data show that some genes involved in proinflammatory or autophagic response are of significantly greater abundance in TS^SYN^ compared to TS^CYT^ in both mock-treated and infected cells, including *PAPPA*, *CARD17*, *TREM1*, *TMED7-TICAM2*, *BATF*, *SLC11A1*, *IL27, ISG20*, *MAK8 IP2*, *MAP1LC3B*, *LAMP3*, *IL16*, *CAPSP1P2*, *CASP4*, *TRIM55*, *ARNT*, and *EIF2AK2* (Fig. 7E).

Using GSEA on our RNA-seq data set comparing TS^SYN^ mock vs TS^CYT^ mock identified 23 “Hallmark” pathway gene sets (FDR-*q* value < 0.01; Fig. 7F). Interestingly, the pathways IFNα*-* and IFNγ-response were both significantly enriched in uninfected (“mock”) TS^SYN^ compared to uninfected TS^CYT^. All the other significant pathways are significantly downregulated in TS^SYNs^ compared to TS^CYTs^, for example, fatty acid metabolism, MTORC1 signaling, apical surface, glycolysis, P53 pathway, epithelial mesenchymal transition, MYC target V1 and V2, apical junction, and E2F targets (Fig. 7F). This is consistent with a distinct response of these cell types to infection as would be expected given the dramatic differences in their transcriptional profiles (Fig. 7A and D).

### TS^SYNs^ recapitulate resistance to *L. monocytogenes* infection compared to TS^CYTs^

**Fig 8:**
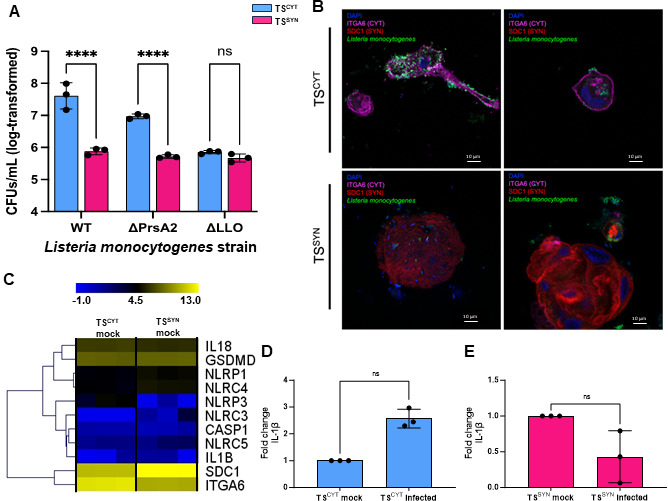
TS^SYNs^ are resistant to *L. monocytogenes* compared to TS^CYTs^. Cells were infected with wild-type (10403S) strain and isogenic ΔprsA2 (NF-L1651) and Δhly (DP-L2161) of *L. monocytogenes* for 8 h. (**A**) CFUs detected on BHI agar plates after 8 h of Lm infection in TS^CYT^ and TS^SYN^. All data are log-transformed for visualization and analysis. For all, * = *P* < 0.05, ** = *P* < 0.01, *** = *P* < 0.001, **** = *P* < 0.0001, and ns = not significant. Data are analyzed with a two-way ANOVA with Holm-Šidák multiple comparisons test. Lm strain, cell type, and interaction are all *P* < 0.0001. Only pre-planned comparisons were made to minimize Type I error, and those are shown on the graph. (**B**) Representative confocal images of GFP-tagged Lm-infected TS^CYT^ or TS^SYN^ for 8 h. ITGA6 (TS^CYT^), SDC1 (TS^SYN^), DAPI (nuclei), and GFP (Lm). Two experiments in three replicates were performed. (**C**) Heat map showing transcript abundance of uninfected TS^CYT^ and TS^SYN^ for genes involved in the inflammasome pathway to illustrate the relatively lower expression of these transcripts compared ITGA6 and SDC1 as representative markers CYTs and SYNs. (**D**) Quantification of the abundance of IL-1β transcript using qPCR normalizing to β-actin as a control. Differences between TS^CYT^ mock and TS^CYT^ infected, and TS^SYN^ mock and TS^SYN^ infected were analyzed by *t* test showing no significant induction of transcript in either Lm-exposed TS^SYNs^ or TS^CYTs^. Two experiments with three replicates were performed.

In an effort to characterize how TS^SYN^ resists the infection of other teratogens besides *T. gondii*, we tested the susceptibility of the cells to the congenital pathogen *L. monocytogenes* (*Lm*). When infected with WT *Lm*, far fewer colony-forming units (CFUs) are recovered from TS^SYN^ compared to TS^CYT^ (Fig. 8A). We observed the same deficit in recovered CFUs when TS^SYN^ and TS^CYT^ infected with *Lm* Δ*prsA2*. PrsA2 is a secreted protein chaperone that is required for the activity of several secreted *Lm* virulence factors and, here, produces an intermediate phenotype (Fig. 8A) (37, 38). Finally, there is no significant difference in CFUs recovered from TS^SYNs^ compared to TS^CYTs^ infected with *Lm* Δ*hly* (encodes listeriolysin O, LLO). LLO is a pore-forming toxin that is required for bacterial escape from the host cell vacuole which allows for intracellular growth and infection of neighboring cells (Fig. 8A) (39). The use of three *Lm* genotypes here depicts a spectrum of permissiveness in TS^CYTs^, whereas TS^SYNs^ entirely restrict *Lm* infection and persistence no matter what *Lm* genotype.

Confocal imaging of GFP-tagged *Lm* infecting either TS^CYT^ or TS^SYN^ (Fig. 8B) mirrors the differences in recovered CFUs depicted for the WT strain (Fig. 8A). We see more *Lm* attached and internalized by TS^CYT^ compared to TS^SYN^. The *Lm* located in TS^SYN^ appears primarily associated with the cell membrane but not intracellular. This distinction is seen clearly in Video S1 as there is no visible intracellular *Lm* in the 3D reconstruction of TS^SYN^ infected with GFP-tagged *Lm*. In TS^CYT^, there are more GFP-tagged *Lm* both associated with the membrane and intracellular. This is visible in Video S2 of TS^CYT^ infected with GFP-tagged *Lm*, especially in contrast to TS^SYN^.

Previous work has shown that the placental trophoblast constitutively secretes the inflammasome cytokines as IL-1β and IL-18, and the infection with *L. monocytogenes* can also induce more activation of this pathway, leading to resistance against the bacteria infection (23). Due to that, we evaluated the gene expression level of some important constituents of the inflammasome pathway, and we identified those genes are expressed in low abundance in both cells TS^CYTs^ and TS^SYNs^ (Fig. 8C). We also measured the gene expression level of IL-1β in infected cells with *L. monocytogenes*, and the infection in both cells does not induce the gene expression of this cytokine when compared to the respective mock conditions (Fig. 8D and E).

**Fig 9:**
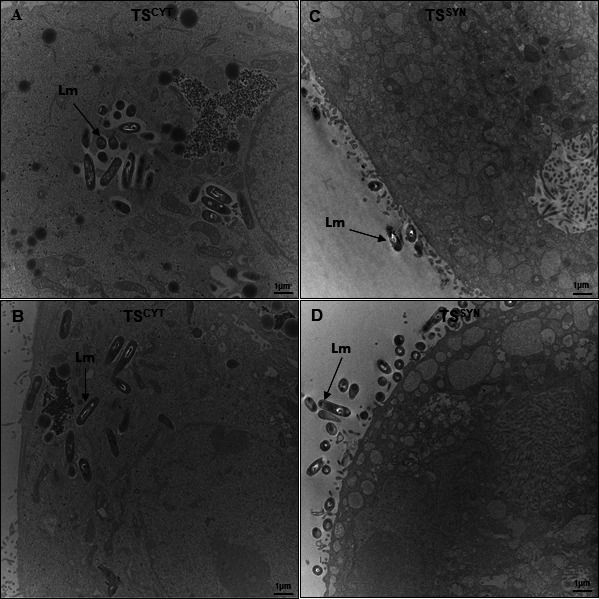
TEM in infected TS^CYTs^ and TS^SYNs^ with *L. monocytogenes.* TS^CYTs^ and TS^SYNs^ were infected with *L. monocytogenes* (WT) for 8 h and processed for TEM. (**A and B**) show the intracellular *L. monocytogenes* in TS^CYTs^. (**C and D**) show *L. monocytogenes* associated with the TS^SYNs^ membrane. Lm: *Listeria monocytogenes*. Black arrows indicate the bacteria. Magnification: 10,000×, scale bar: 1 µm.

We also performed TEM in TS^CYTs^ and TS^SYNs^ infected with *Lm*. The representative EM photos confirm the data found in the confocal imaging (Fig. 8B), in which most of the *Lm* was associated to the cell membrane of TS^SYNs^; however, in TS^CYTs^, *Lm* are successfully internalized and grow intracellularly (Fig. 9A through D).

## DISCUSSION

Here, we describe a new *in vitro* system developed (27) to study placenta-pathogen interactions evaluating the differential susceptibility of TS^SYNs^ and TS^CYTs^ against *T. gondii*. The architecture of placenta villous explants, comprised primarily of trophoblast cells, is associated with safeguarding the fetus against potential maternal bloodborne microbes, but the mechanisms underlying the resistance profile are still poorly understood (2, 16).

Nearly all mammalian cells studied to date can be infected with *T. gondii* and support rapid *T. gondii* replication unless they are made resistant by exposure to effector cytokines like interferon-γ. However, placental syncytiotrophoblasts are an exception to this rule, having clear innate resistance to *T. gondii* that has been demonstrated in multiple primary placental models including villous explants and PHT cells (18, 24). The mechanisms involved in SYN resistance to parasite adhesion and ability to restrict parasite growth are unknown (16, 18), and a significant barrier to elucidating these mechanisms is genetically tractable and reproducible models of CYT and SYN development. Overall, our data provide evidence that TS-derived CYTs and SYNs recapitulate the susceptibility and resistance phenotypes that we and others have previously characterized. Most notably, TS^SYNs^ resist *T. gondii* infection at the level of both adhesion and replication, which is identical to what we have observed previously using mixed CYT and SYN cultures derived from term placentas (18). This same dichotomy is observed in the midgestational chorionic villous explants (18, 40). Additionally, another study using villous explants from the first trimester showed that SYN acts as a strong barrier against *T. gondii* since these cells were also very resistant to infection (24).

Mechanisms of SYN resistance to a variety of pathogens seem to depend on the infection model. Our data showed that besides the resistance against *T. gondii* infection, TS^SYNs^ also were resistant to *L. monocytogenes*, mainly by restricting bacterial entry into the cells. Looking at *L. monocytogenes* and *T. gondii* infection, we can observe that TS^SYNs^ are very resistant to pathogen invasion/entry, similar to what is shown in SYNs from PHTs and villous explants (18, 22, 41). SYNs lack intracellular junctions, which some bacterial and viral pathogens use to invade cells (42, 43). They also have a robust cytoskeletal network and branched microvilli that might inhibit pathogen entry and in fact are highly resistant to *T. gondii* adhesion, a required first step for *T. gondii* to ultimately infect a mammalian cell (18). Here, our work shows a clear difference in the density of the microvilli between TS^SYNs^ and TS^CYTs^, in which the cell surface of TS^SYNs^ is highly density covered by microvilli while in TS^CYTs^ is not. SEM photos from both infected cells for 2 h clearly show more parasites that invade TS^CYTs^ (Fig. 5A through D), and in TS^SYNs^, *T. gondii* seems to be attached to the membrane but rarely invades the cells in the same time frame as compared to TS^CYTs^ (Fig. 5E through G). Interestingly, besides the presence of microvilli, TS^SYNs^ have a significant decrease in the transcriptome abundance of a large number of genes related to apical surface and apical junctions, including *ICAM1*, *TJP1*, *CDH1*, and *CDH3*, that are described to be important for pathogens invasion, sharing the same features as SYNs from PHTs cells (Fig. S2A). So, we suggest that these features could be involved in the differential invasion/entry rates of *T. gondii* and *L. monocytogenes* in TS^CYTs^ compared to TS^SYNs^. Surface proteoglycan content may also differ in TS^SYN^ compared to TS^CYT^, a possible mechanism supported by both RNA-seq data in the present study and others (27) that TS^SYN^ has lower transcript abundance for HSPG2 and ICAM-1 compared to TS^CYT^ (Table S1). HSPG2 and ICAM-1 are involved in *T. gondii* attachment and invasion, being targeted by parasite surface proteins such and SAG-3 and MIC-2, respectively, to promote invasion (44, 45). Host cell surface proteoglycans are also generally critical for *T. gondii* adhesion (46), and our transcriptional data also show a clear reduction in the levels of XYLT1 transcript during the TS^CYT^ to TS^SYN^ conversion *in vitro* (Table S1), which catalyzes one of the first steps in proteoglycan synthesis by adding xylose to serine residues in target proteins (47). This is another possible means of restricting pathogen adhesion to SYNs, in particular for those that require preliminary adhesion events to proteoglycans. Lectin-based studies have also demonstrated clear differences in surface sugar content across different placental cell types including CYTs and SYNs (48). The TS^SYN^ and TS^CYT^ system could be used to study the role of surface proteoglycan content on cell-specific restriction in *T. gondii/L. monocytogenes* adhesion given its reproducible growth and differentiation characteristics and genetic tractability.

In addition to its physical barrier function, the trophoblast triggers a powerful immune response by releasing various cytokines and immunological factors. Trophoblasts produce cytokines constitutively and in response to infection, including those associated with the inflammasome, such as IL-1β and IL-18, which control *L. monocytogenes* infection (23). In contrast, CCL22 is only detected in large quantities following infection of PHT cells with *T. gondii* (18, 28) and in trophoblast organoids after infection. CCL22 also increases in abundance during miscarriage (49, 50). One interesting data is the increase of MIF in TS^SYNs^ when compared to TS^CYTs^, especially after infection. MIF is extensively reported to be produced by different placenta models such as BeWo cells and villous explants, and its production has been related to controlling the proliferation of *T. gondii* (51–53). Our transcriptome data in TS cells do not show an increase in MIF gene expression after infection. Due to that, we can suggest that this increase might be regulated at the post-transcriptional level and the role of MIF in controlling *T. gondii* proliferation in TS^SYNs^ should be investigated. In general, our data showed that both TS^CYTs^ and TS^SYNs^ do not recapitulate the immunological secretome previously observed in PHT cells and villous explants and in the present placental organoids (Fig. 6F, G and H). Therefore, with respect to *T. gondii*, at least, TS^SYNs^ can be used to directly explore the structural impediments to parasite adhesion and mechanisms of IFNγ-independent restriction of parasite replication, while other models like the placental organoids are more useful for studying both basal and induced immunological mechanisms of resistance.

The TS model permits us to circumvent one limitation of the PHT model which is that the cultures are a mixture of both CYT and SYN cells that vary in their ratios between preparations. Given that TS-derived CYTs and SYNs can be cultivated in a manner that leads to relatively pure cultures of a given cell type, we could examine putative CYT- and SYN-specific responses in isolation. We observed large differences in the transcriptional responses of each cell type, but one of the more remarkable findings was that although *T. gondii* invaded and proliferated poorly in TS^SYNs^, we still observed considerable changes in the transcriptional profile of these cells that rivaled those found in the more readily infected TS^CYTs^. The changes that we observed in the TS^SYN^ could be driven more by paracrine responses to the presence of *T. gondii* rather than infection, although this would have to be investigated directly. It is also possible that the TS^SYN^ is particularly sensitive to alterations induced by even the small number of invaded parasites, and/or resistance pathways that drive the clear phenotype of restricting *T. gondii* replication by the TS^SYN^ are robustly activated by *T. gondii*.

Human trophoblast stem cells have emerged in recent years as an important tool in studying placental development as well as pathogen resistance and responses. Here, we show that these cells recapitulate primary human trophoblast and explant resistance phenotype profiles, with TS-derived SYNs being highly resistant to *T. gondii* infection and being ultra structurally similar to primary cells. TS-derived SYNs also resisted infection with *L. monocytogenes* [a feature shared with placental explants (22, 23)], suggesting that resisting pathogen adhesion/attachment may be a generalized mechanism of SYN resistance. However, the TS model has some limitations, most notably in its poor recapitulation of both constitutive and pathogen-induced cytokine production which is observed in primary trophoblast cultures and placental explants (18). These cells are genetically tractable tools to investigate cell-intrinsic mechanisms of resistance to pathogen adhesion and replication.

## Data Availability

Data have been deposited in NCBI’s Gene Expression Omnibus and are accessible through GEO Series accession number GSE263615).

## SUPPLEMENTAL MATERIAL

Fig. S1 msphere.00748-23-s0001.tif Transmission electron microscopy in TSCYTs and TSSYNs.

Fig. S2 msphere.00748-23-s0002.tif Apical surface and apical junction gene set.

Supplemental legends msphere.00748-23-s0003.docx Legends for Fig. S1 and S2, Videos S1 and 2, and Table S1.

Table S1 msphere.00748-23-s0004.xlsx Log2 (FPKM) transcript count values from TSSYNs and TSCYTs mock and infected with *Toxoplasma gondii*.

Video S1 msphere.00748-23-s0005.mp4 3D reconstruction of TSCYT infected with GFP-tagged Lm.

Video S2 msphere.00748-23-s0006.mp4 3D reconstruction of TSSYN infected with GFP-tagged Lm.

ASM does not own the copyrights to Supplemental Material that may be linked to, or accessed through, an article. The authors have granted ASM a non-exclusive, world-wide license to publish the Supplemental Material files. Please contact the corresponding author directly for reuse.
