# Supplementary figures and images for: The trophoblast surface becomes refractory to adhesion by congenitally transmitted Toxoplasma gondii and Listeria monocytogenes during cytotrophoblast to syncytiotrophoblast development
Source: mSphere. 2024 May 21;9(6):e00748-23. doi: 10.1128/msphere.00748-23 (PMC11332349; doi:10.1128/msphere.00748-23)

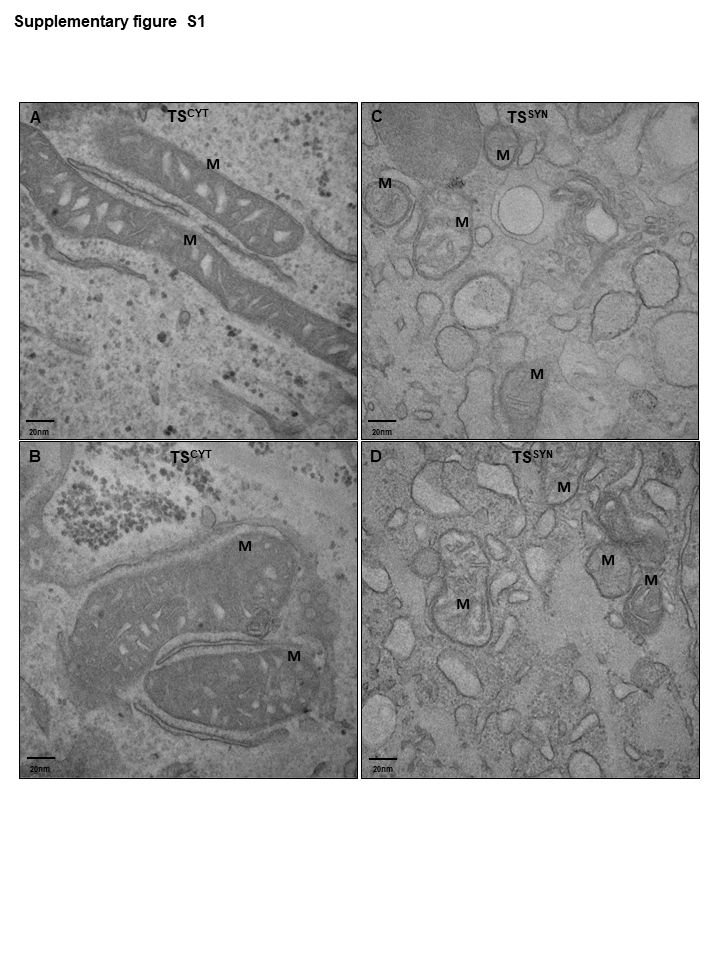

Supplement: Fig. S1 — Transmission electron microscopy in TSCYTs and TSSYNs. [file msphere.00748-23-s0001.tif]

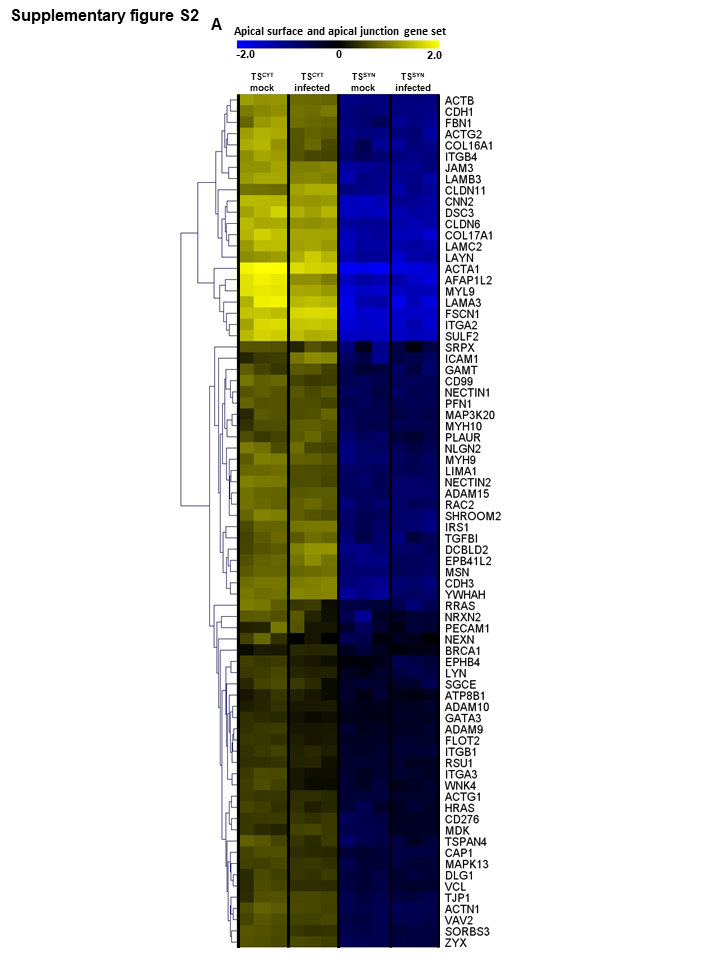

Supplement: Fig. S2 — Apical surface and apical junction gene set. [file msphere.00748-23-s0002.tif]
