# Supplementary material for: The trophoblast surface becomes refractory to adhesion by congenitally transmitted Toxoplasma gondii and Listeria monocytogenes during cytotrophoblast to syncytiotrophoblast development
Source: mSphere. 2024 May 21;9(6):e00748-23. doi: 10.1128/msphere.00748-23 (PMC11332349; doi:10.1128/msphere.00748-23)
Supplement: Supplemental legends — Legends for Fig. S1 and S2, Videos S1 and 2, and Table S1. [file msphere.00748-23-s0003.docx]

**Supplementary figure S1. Transmission electron microscopy in TS^CYTs^ and TS^SYNs^.** TS^CYT^ and TS^SYN^ were processed for TEM to differences in cells organelles in both cells. Representative TEM images show the mitochondria in TS^CYTs^ (**A** and **B**) and in TS^SYNs^ **(C** and **D**). Mitochondria= M. Mag. 60,000x, scale bar: 200nm.

**Supplementary figure S2. Apical surface and apical junction genes set.** Using the bulk RNAseq data generated by TS^CYTs^ and TS^SYNs^ mock and infected with *T. gondii* we used the Pre-ranked Gene Set Enrichment Analysis (GSEA) from rlog-normalized data to evaluate the differences in enriched pathways between TS^SYN^ and TS^CYT^*.* Among the gene sets that are significantly downregulated in TS^SYNs^ (FDR q-value < 0.05), we evaluated the apical surface and apical junction genes. (**A**) Heat map showing the apical surface and apical junction gene sets that have significantly different transcript abundance between TS^CYTs^ and TS^SYNs^.

**Supplementary video 1**. **3D reconstruction of TS^CYT^ infected with GFP-tagged *Lm.*** TS^CYTs^ were infected with GFP-tagged *Lm* for 8 hours and cells were analyzed by confocal microscopy. Images in z-stack were made to develop the 3D reconstruction. TS^CYT^ marker (ITGA-6) (pink), DAPI (nuclei) and *Lm* GFP (green).

**Supplementary video 2**. **3D reconstruction of TS^SYN^ infected with GFP-tagged *Lm.*** TS^SYNs^ were infected with GFP-tagged *Lm* for 8 hours and cells were analyzed by confocal microscopy. Images in z-stack were made to develop the 3D reconstruction. TS^SYN^ marker SDC1 (red), DAPI (nuclei) and *Lm* GFP (green).

**Supplementary table S1: Log2 (FPKM) transcript count values from TS^SYNs^ and TS^CYTs^ mock and infected with *Toxoplasma gondii*.**
